# Methyl 4-(4-chloro­phen­yl)-3,3a,4,4a,5,12c-hexa­hydro-2-thia­naphtho­[1′,2′:3,2]furo[5,4-*b*]pyrrolizine-4a-carboxyl­ate

**DOI:** 10.1107/S1600536810028746

**Published:** 2010-07-24

**Authors:** S. Selvanayagam, B. Sridhar, K. Ravikumar, S. Kathiravan, R. Raghunathan

**Affiliations:** a Department of Physics, Kalasalingam University, Krishnankoil 626 190, India; b Laboratory of X-ray Crystallography, Indian Institute of Chemical Technology, Hyderabad 500 007, India; c Department of Organic Chemistry, University of Madras, Guindy Campus, Chennai 600 025, India

## Abstract

In the title compound, C_25_H_22_ClNO_3_S, both the pyrrolidinyl and thia­zolyl rings adopt envelope conformations whereas the dihydro­pyran ring adopts a half-chair conformation. The chloro­phenyl and naphthalenyl ring systems are oriented at a dihedral angle of 59.7 (1)°. The crystal packing is stabilized by an intra­molecular C—H⋯N hydrogen bond and weak inter­molecular C—H⋯π inter­actions.

## Related literature

For related structures, see: Nirmala *et al.* (2009 ▶); Selvanayagam *et al.* (2010 ▶). For the superposition of related structures, see: Gans & Shalloway (2001 ▶). For ring-puckering and asymmetry parameters, see: Cremer & Pople (1975 ▶); Nardelli (1983 ▶).
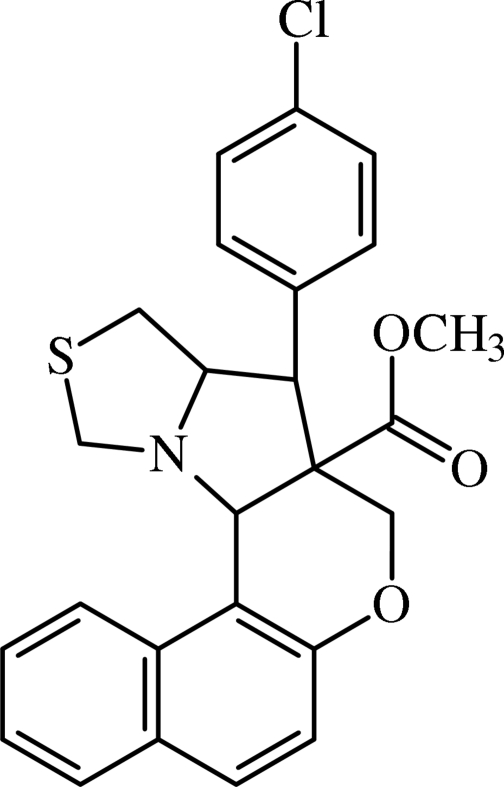


## Experimental

### 

#### Crystal data

C_25_H_22_ClNO_3_S
*M* *_r_* = 451.95
Orthorhombic, 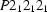

*a* = 8.0740 (6) Å
*b* = 12.1109 (8) Å
*c* = 22.1813 (15) Å
*V* = 2169.0 (3) Å^3^
*Z* = 4
Mo *K*α radiation
μ = 0.30 mm^−1^
*T* = 292 K
0.23 × 0.21 × 0.19 mm

#### Data collection

Bruker SMART APEX CCD area-detector diffractometer
25236 measured reflections
5157 independent reflections
4559 reflections with *I* > 2σ(*I*)
*R*_int_ = 0.024

#### Refinement

*R*[*F* ^2^ > 2σ(*F* ^2^)] = 0.044
*wR*(*F* ^2^) = 0.110
*S* = 1.04
5157 reflections
281 parameters
H-atom parameters constrained
Δρ_max_ = 0.28 e Å^−3^
Δρ_min_ = −0.15 e Å^−3^
Absolute structure: Flack (1983 ▶), 2186 Friedel pairs
Flack parameter: 0.01 (6)

### 

Data collection: *SMART* (Bruker, 2001 ▶); cell refinement: *SAINT* (Bruker, 2001 ▶); data reduction: *SAINT*; program(s) used to solve structure: *SHELXS97* (Sheldrick, 2008 ▶); program(s) used to refine structure: *SHELXL97* (Sheldrick, 2008 ▶); molecular graphics: *ORTEP-3* (Farrugia, 1997 ▶) and *PLATON* (Spek, 2009 ▶); software used to prepare material for publication: *SHELXL97* and *PLATON*.

## Supplementary Material

Crystal structure: contains datablocks I, global. DOI: 10.1107/S1600536810028746/ng5001sup1.cif

Structure factors: contains datablocks I. DOI: 10.1107/S1600536810028746/ng5001Isup2.hkl

Additional supplementary materials:  crystallographic information; 3D view; checkCIF report

## Figures and Tables

**Table 1:** Hydrogen-bond geometry (Å, °) *Cg* is the centroid of the chloro­phenyl ring.

*D*—H⋯*A*	*D*—H	H⋯*A*	*D*⋯*A*	*D*—H⋯*A*
C12—H12*B*⋯N1	0.97	2.57	2.903 (3)	100
C25—H25*A*⋯*Cg*^i^	0.96	2.79	3.431 (3)	125

Symmetry code: (i) 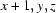.

**Fig. 1.**
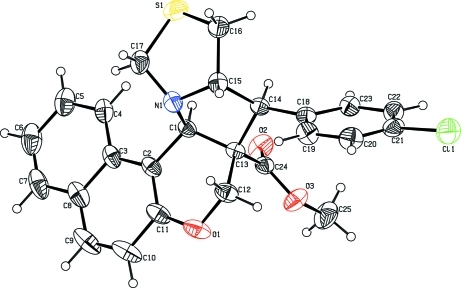
The molecular structure of the title compound, showing the atom-numbering scheme. Displacement ellipsoids are drawn at the 30% probability level

**Fig. 2.**
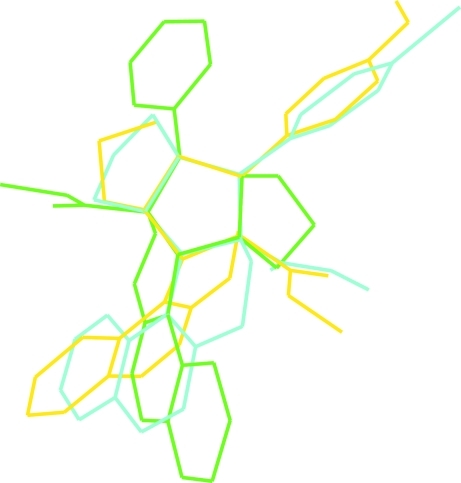
Superposition of (I) (cyan) with the similar reported structures of Nirmala et al. (2009) (yellow) and Selvanayagam et al. (2010).

**Fig. 3.**
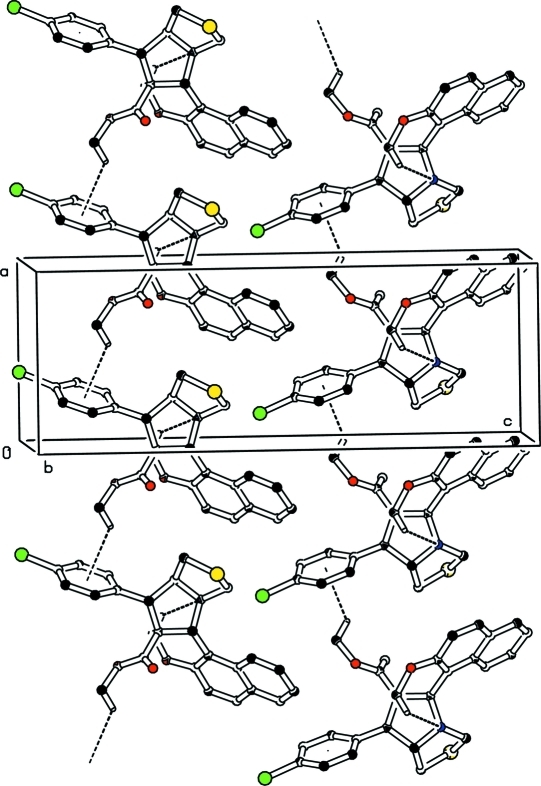
Molecular packing of the title compound, viewed along the b axis.

## supplementary crystallographic information

### Comment

In continuation of our work on the crystal structure analysis of pyrrolizine derivatives, we have undertaken a single-crystal X-ray diffraction study for the title compound, and the results are presented here.

The X-ray study confirmed the molecular structure and atomic connectivity for (I), as illustrated in Fig. 1. A l l the bond lengths are normal and comparable to the standard values. Fig. 2 shows a superposition of the pyrrolidine ring of (I) with the related reported structures of Nirmala *et al.* (2009) and Selvanayagam *et al.* (2010), using Qmol (Gans & Shalloway, 2001); the r.m.s. deviation is 0.350 and 0.864 Å, respectively. The sum of the angles (331.9°) around atom N1 is in accordance with *sp*^3^ hybridization.

The chlorine atom deviates 0.118 (1) Å from the best plane of chlorophenyl ring. The naphthalene ring system (C2–C11) and the chlorophenyl ring are oriented with a dihedral angle of 59.7 (1)°. In the thiapyrrolizine ring system, both the pyrrolidine and thiazole rings N1/C1/C13–C15 and N1/C15/C16/S1/C17 adopt envelope conformations; the puckering parameters (Cremer & Pople, 1975) are: q_2_ = 0.447 (2) Å and φ = -118.7 (2)° for N1/C1/C13–C15 ring, and q_2_ = 0.508 (2) Å and φ = -59.9 (2)° for N1/C15/C16/S1/C17 ring. In the N1/C1/C13–C15 ring, atom C13 deviates by -0.675 (2) Å from the least-squares plane through the remaining four atoms, whereas in the ring N1/C15/C16/S1/C17, atom S1 deviates by -0.855 (1) Å from the plane through the remaining four atoms. The dihydropyran ring of the chromene unit adopts a half-chair conformation, with the lowest asymmetry parameter ΔC_2_(C2–C11) of 0.039 (1)° (Nardelli, 1983).

In addition to van der Waals interactions, the molecular packing is stabilized by intramolecular C—H···N hydrogen bond and intermolecular weak C—H···π interactions (Fig. 3).

### Experimental

A mixture of (*Z*)-methyl-2[(1-formylnaphthalen-2-yloxy)methyl]-3-(4-chlorophenyl) acrylate (20 mmol) and thiaproline (30 mmol) was refluxed in benzene for 20 h and the solvent was removed under reduced pressure. The crude product was subjected to column chromatography to get the pure product. Single crystals were grown by slow evapoartion of a chloroform-methanol (1:1) soution.

### Refinement

H atoms were placed in idealized positions and allowed to ride on their parent atoms, with C—H = 0.93, 0.98, 0.97 and 0.96 Å for aromatic, methine, methylene and methyl H respectively, and *U*_iso_(H) = 1.5*U*_eq_(C) for methyl H and *U*_iso_(H) = 1.2*U*_eq_(C) for all other H atoms.

### Crystal data

**Table:**

C_25_H_22_ClNO_3_S	*F*(000) = 944
*M**_r_* = 451.95	*D*_x_ = 1.384 Mg m^−^^3^
Orthorhombic, *P*2_1_2_1_2_1_	Mo *K*α radiation, λ = 0.71073 Å
Hall symbol: P 2ac 2ab	Cell parameters from 16148 reflections
*a* = 8.0740 (6) Å	θ = 2.1–27.6°
*b* = 12.1109 (8) Å	µ = 0.30 mm^−^^1^
*c* = 22.1813 (15) Å	*T* = 292 K
*V* = 2169.0 (3) Å^3^	Block, colourless
*Z* = 4	0.23 × 0.21 × 0.19 mm

### Data collection

**Table:**

Bruker SMART APEX CCD area-detector diffractometer	4559 reflections with *I* > 2σ(*I*)
Radiation source: fine-focus sealed tube	*R*_int_ = 0.024
graphite	θ_max_ = 28.1°, θ_min_ = 1.8°
ω scans	*h* = −10→10
25236 measured reflections	*k* = −15→16
5157 independent reflections	*l* = −29→29

### Refinement

**Table:**

Refinement on *F*^2^	Secondary atom site location: difference Fourier map
Least-squares matrix: full	Hydrogen site location: inferred from neighbouring sites
*R*[*F*^2^ > 2σ(*F*^2^)] = 0.044	H-atom parameters constrained
*wR*(*F*^2^) = 0.110	*w* = 1/[σ^2^(*F*_o_^2^) + (0.0624*P*)^2^ + 0.2403*P*] where *P* = (*F*_o_^2^ + 2*F*_c_^2^)/3
*S* = 1.04	(Δ/σ)_max_ = 0.001
5157 reflections	Δρ_max_ = 0.28 e Å^−^^3^
281 parameters	Δρ_min_ = −0.15 e Å^−^^3^
0 restraints	Absolute structure: Flack (1983), 2186 Friedel pairs
Primary atom site location: structure-invariant direct methods	Flack parameter: 0.01 (6)

### Special details

**Table:**

Geometry. All e.s.d.'s (except the e.s.d. in the dihedral angle between two l.s. planes) are estimated using the full covariance matrix. The cell e.s.d.'s are taken into account individually in the estimation of e.s.d.'s in distances, angles and torsion angles; correlations between e.s.d.'s in cell parameters are only used when they are defined by crystal symmetry. An approximate (isotropic) treatment of cell e.s.d.'s is used for estimating e.s.d.'s involving l.s. planes.
Refinement. Refinement of *F*^2^ against ALL reflections. The weighted *R*-factor *wR* and goodness of fit *S* are based on *F*^2^, conventional *R*-factors *R* are based on *F*, with *F* set to zero for negative *F*^2^. The threshold expression of *F*^2^ > σ(*F*^2^) is used only for calculating *R*-factors(gt) *etc*. and is not relevant to the choice of reflections for refinement. *R*-factors based on *F*^2^ are statistically about twice as large as those based on *F*, and *R*- factors based on ALL data will be even larger.

### Fractional atomic coordinates and isotropic or equivalent isotropic displacement parameters (Å^2^)

**Table:**

	*x*	*y*	*z*	*U*_iso_*/*U*_eq_	
Cl1	−0.39547 (9)	0.77136 (7)	0.50917 (3)	0.0888 (2)	
S1	−0.28910 (8)	1.01078 (6)	0.12895 (3)	0.07732 (19)	
O1	0.2032 (2)	0.67004 (11)	0.21872 (8)	0.0728 (5)	
O2	0.2227 (2)	0.98332 (12)	0.25861 (7)	0.0698 (4)	
O3	0.21864 (19)	0.84359 (14)	0.32261 (7)	0.0693 (4)	
N1	−0.13547 (18)	0.82523 (13)	0.15571 (7)	0.0510 (4)	
C1	0.03210 (19)	0.86266 (14)	0.17424 (8)	0.0409 (3)	
H1	0.0406	0.9429	0.1697	0.049*	
C2	0.1710 (2)	0.80726 (14)	0.14034 (9)	0.0479 (4)	
C3	0.2357 (2)	0.85149 (16)	0.08546 (9)	0.0514 (4)	
C4	0.1812 (3)	0.95119 (18)	0.05993 (8)	0.0586 (5)	
H4	0.1006	0.9921	0.0799	0.070*	
C5	0.2432 (3)	0.9901 (3)	0.00649 (10)	0.0750 (6)	
H5	0.2045	1.0563	−0.0094	0.090*	
C6	0.3656 (3)	0.9295 (3)	−0.02419 (11)	0.0887 (9)	
H6	0.4055	0.9545	−0.0611	0.106*	
C7	0.4251 (3)	0.8353 (3)	−0.00020 (12)	0.0814 (8)	
H7	0.5082	0.7971	−0.0204	0.098*	
C8	0.3646 (3)	0.79322 (19)	0.05482 (11)	0.0647 (6)	
C9	0.4312 (3)	0.6989 (2)	0.08106 (14)	0.0822 (8)	
H9	0.5146	0.6606	0.0611	0.099*	
C10	0.3768 (3)	0.66178 (18)	0.13531 (15)	0.0802 (7)	
H10	0.4270	0.6010	0.1532	0.096*	
C11	0.2438 (2)	0.71561 (15)	0.16450 (11)	0.0596 (5)	
C12	0.0538 (3)	0.70803 (15)	0.24674 (11)	0.0570 (5)	
H12A	0.0551	0.6882	0.2891	0.068*	
H12B	−0.0405	0.6720	0.2281	0.068*	
C13	0.0355 (2)	0.83233 (13)	0.24070 (8)	0.0401 (3)	
C14	−0.1375 (2)	0.87464 (13)	0.26073 (8)	0.0416 (3)	
H14	−0.1306	0.9554	0.2608	0.050*	
C15	−0.2488 (2)	0.84365 (17)	0.20738 (8)	0.0511 (4)	
H15	−0.3079	0.7750	0.2166	0.061*	
C16	−0.3753 (2)	0.9351 (2)	0.19154 (10)	0.0695 (6)	
H16A	−0.3922	0.9837	0.2258	0.083*	
H16B	−0.4809	0.9028	0.1804	0.083*	
C17	−0.2033 (3)	0.8788 (2)	0.10353 (9)	0.0636 (5)	
H17A	−0.2894	0.8336	0.0856	0.076*	
H17B	−0.1175	0.8910	0.0736	0.076*	
C18	−0.1970 (2)	0.84189 (14)	0.32279 (8)	0.0447 (4)	
C19	−0.2712 (3)	0.74099 (17)	0.33593 (10)	0.0611 (5)	
H19	−0.2815	0.6881	0.3058	0.073*	
C20	−0.3297 (3)	0.71840 (19)	0.39317 (11)	0.0664 (6)	
H20	−0.3805	0.6512	0.4014	0.080*	
C21	−0.3120 (3)	0.79610 (19)	0.43776 (9)	0.0585 (5)	
C22	−0.2352 (3)	0.89380 (18)	0.42702 (9)	0.0594 (5)	
H22	−0.2210	0.9450	0.4579	0.071*	
C23	−0.1785 (2)	0.91603 (15)	0.36953 (9)	0.0507 (4)	
H23	−0.1262	0.9831	0.3621	0.061*	
C24	0.1702 (2)	0.89594 (14)	0.27369 (8)	0.0441 (4)	
C25	0.3441 (3)	0.8996 (3)	0.35799 (12)	0.0927 (9)	
H25A	0.4469	0.8997	0.3363	0.139*	
H25B	0.3584	0.8619	0.3957	0.139*	
H25C	0.3099	0.9743	0.3654	0.139*	

### Atomic displacement parameters (Å^2^)

**Table:**

	*U*^11^	*U*^22^	*U*^33^	*U*^12^	*U*^13^	*U*^23^
Cl1	0.0877 (4)	0.1109 (5)	0.0676 (3)	0.0124 (4)	0.0262 (3)	0.0256 (3)
S1	0.0624 (3)	0.0981 (4)	0.0715 (3)	0.0219 (3)	−0.0083 (3)	0.0120 (3)
O1	0.0696 (9)	0.0436 (7)	0.1053 (12)	0.0169 (7)	0.0198 (9)	0.0080 (7)
O2	0.0752 (10)	0.0577 (8)	0.0764 (9)	−0.0241 (8)	−0.0210 (8)	0.0036 (7)
O3	0.0568 (8)	0.0881 (10)	0.0630 (9)	−0.0065 (8)	−0.0181 (7)	0.0179 (8)
N1	0.0377 (7)	0.0616 (9)	0.0536 (8)	−0.0053 (7)	−0.0014 (6)	−0.0136 (7)
C1	0.0361 (7)	0.0378 (8)	0.0490 (9)	−0.0001 (6)	−0.0001 (7)	−0.0084 (6)
C2	0.0380 (8)	0.0436 (8)	0.0622 (10)	−0.0047 (7)	0.0031 (8)	−0.0183 (8)
C3	0.0396 (9)	0.0584 (10)	0.0560 (10)	−0.0130 (8)	0.0042 (7)	−0.0246 (8)
C4	0.0552 (11)	0.0717 (12)	0.0488 (10)	−0.0096 (10)	0.0044 (8)	−0.0121 (9)
C5	0.0699 (13)	0.1004 (16)	0.0549 (11)	−0.0197 (13)	0.0044 (10)	−0.0034 (12)
C6	0.0671 (15)	0.140 (3)	0.0586 (14)	−0.0266 (18)	0.0179 (12)	−0.0136 (15)
C7	0.0525 (12)	0.117 (2)	0.0743 (15)	−0.0184 (14)	0.0228 (11)	−0.0421 (16)
C8	0.0415 (9)	0.0743 (13)	0.0783 (14)	−0.0148 (10)	0.0118 (9)	−0.0323 (11)
C9	0.0540 (12)	0.0672 (14)	0.125 (2)	−0.0026 (11)	0.0293 (14)	−0.0387 (15)
C10	0.0567 (12)	0.0478 (11)	0.136 (2)	0.0087 (10)	0.0226 (15)	−0.0153 (13)
C11	0.0502 (10)	0.0388 (9)	0.0899 (15)	−0.0004 (8)	0.0112 (10)	−0.0130 (9)
C12	0.0566 (11)	0.0405 (9)	0.0739 (13)	0.0020 (8)	0.0084 (10)	0.0057 (8)
C13	0.0345 (7)	0.0346 (7)	0.0510 (9)	0.0001 (6)	0.0008 (7)	−0.0017 (6)
C14	0.0337 (7)	0.0401 (8)	0.0511 (9)	−0.0012 (6)	0.0019 (7)	−0.0004 (7)
C15	0.0374 (8)	0.0641 (10)	0.0518 (10)	−0.0081 (7)	0.0012 (7)	−0.0060 (8)
C16	0.0389 (10)	0.1115 (17)	0.0580 (11)	0.0141 (11)	−0.0023 (8)	−0.0057 (12)
C17	0.0440 (9)	0.0976 (16)	0.0491 (10)	−0.0024 (11)	−0.0040 (9)	−0.0129 (10)
C18	0.0350 (8)	0.0460 (8)	0.0530 (9)	−0.0001 (7)	0.0004 (7)	0.0019 (7)
C19	0.0650 (12)	0.0503 (10)	0.0680 (12)	−0.0094 (9)	0.0068 (10)	−0.0009 (9)
C20	0.0659 (13)	0.0591 (11)	0.0742 (13)	−0.0068 (10)	0.0101 (11)	0.0146 (10)
C21	0.0465 (10)	0.0742 (12)	0.0547 (10)	0.0105 (10)	0.0059 (8)	0.0164 (9)
C22	0.0547 (11)	0.0701 (12)	0.0535 (10)	0.0023 (10)	−0.0019 (9)	−0.0027 (9)
C23	0.0442 (9)	0.0528 (9)	0.0553 (10)	−0.0042 (7)	−0.0006 (8)	−0.0006 (8)
C24	0.0336 (8)	0.0499 (9)	0.0489 (9)	0.0028 (7)	0.0009 (7)	−0.0022 (7)
C25	0.0586 (14)	0.150 (3)	0.0690 (15)	−0.0026 (16)	−0.0224 (12)	−0.0050 (16)

### Geometric parameters (Å, °)

**Table:**

Cl1—C21	1.7473 (19)	C10—C11	1.413 (3)
S1—C16	1.803 (2)	C10—H10	0.9300
S1—C17	1.831 (2)	C12—C13	1.519 (2)
O1—C11	1.363 (3)	C12—H12A	0.9700
O1—C12	1.433 (3)	C12—H12B	0.9700
O2—C24	1.188 (2)	C13—C24	1.520 (2)
O3—C24	1.316 (2)	C13—C14	1.553 (2)
O3—C25	1.450 (3)	C14—C18	1.511 (2)
N1—C17	1.435 (3)	C14—C15	1.533 (2)
N1—C15	1.484 (2)	C14—H14	0.9800
N1—C1	1.485 (2)	C15—C16	1.547 (3)
C1—C2	1.507 (2)	C15—H15	0.9800
C1—C13	1.519 (2)	C16—H16A	0.9700
C1—H1	0.9800	C16—H16B	0.9700
C2—C11	1.366 (3)	C17—H17A	0.9700
C2—C3	1.429 (3)	C17—H17B	0.9700
C3—C4	1.404 (3)	C18—C23	1.380 (3)
C3—C8	1.429 (3)	C18—C19	1.392 (3)
C4—C5	1.370 (3)	C19—C20	1.382 (3)
C4—H4	0.9300	C19—H19	0.9300
C5—C6	1.406 (4)	C20—C21	1.373 (3)
C5—H5	0.9300	C20—H20	0.9300
C6—C7	1.348 (4)	C21—C22	1.357 (3)
C6—H6	0.9300	C22—C23	1.381 (3)
C7—C8	1.410 (4)	C22—H22	0.9300
C7—H7	0.9300	C23—H23	0.9300
C8—C9	1.390 (4)	C25—H25A	0.9600
C9—C10	1.358 (4)	C25—H25B	0.9600
C9—H9	0.9300	C25—H25C	0.9600
			
C16—S1—C17	86.54 (11)	C24—C13—C14	109.79 (13)
C11—O1—C12	117.08 (16)	C18—C14—C15	116.91 (14)
C24—O3—C25	115.36 (19)	C18—C14—C13	117.39 (14)
C17—N1—C15	108.65 (15)	C15—C14—C13	103.06 (14)
C17—N1—C1	115.64 (16)	C18—C14—H14	106.2
C15—N1—C1	107.59 (13)	C15—C14—H14	106.2
N1—C1—C2	113.81 (13)	C13—C14—H14	106.2
N1—C1—C13	102.20 (14)	N1—C15—C14	105.76 (13)
C2—C1—C13	111.28 (14)	N1—C15—C16	109.84 (16)
N1—C1—H1	109.8	C14—C15—C16	112.78 (16)
C2—C1—H1	109.8	N1—C15—H15	109.5
C13—C1—H1	109.8	C14—C15—H15	109.5
C11—C2—C3	118.79 (17)	C16—C15—H15	109.5
C11—C2—C1	119.09 (17)	C15—C16—S1	106.49 (13)
C3—C2—C1	122.02 (16)	C15—C16—H16A	110.4
C4—C3—C2	123.46 (17)	S1—C16—H16A	110.4
C4—C3—C8	117.4 (2)	C15—C16—H16B	110.4
C2—C3—C8	119.1 (2)	S1—C16—H16B	110.4
C5—C4—C3	122.0 (2)	H16A—C16—H16B	108.6
C5—C4—H4	119.0	N1—C17—S1	106.90 (13)
C3—C4—H4	119.0	N1—C17—H17A	110.3
C4—C5—C6	119.7 (3)	S1—C17—H17A	110.3
C4—C5—H5	120.1	N1—C17—H17B	110.3
C6—C5—H5	120.1	S1—C17—H17B	110.3
C7—C6—C5	120.1 (2)	H17A—C17—H17B	108.6
C7—C6—H6	120.0	C23—C18—C19	117.43 (17)
C5—C6—H6	120.0	C23—C18—C14	118.64 (15)
C6—C7—C8	121.6 (2)	C19—C18—C14	123.92 (16)
C6—C7—H7	119.2	C20—C19—C18	120.9 (2)
C8—C7—H7	119.2	C20—C19—H19	119.6
C9—C8—C7	121.7 (2)	C18—C19—H19	119.6
C9—C8—C3	119.2 (2)	C21—C20—C19	119.39 (19)
C7—C8—C3	119.0 (2)	C21—C20—H20	120.3
C10—C9—C8	121.2 (2)	C19—C20—H20	120.3
C10—C9—H9	119.4	C22—C21—C20	121.25 (18)
C8—C9—H9	119.4	C22—C21—Cl1	119.01 (18)
C9—C10—C11	119.9 (2)	C20—C21—Cl1	119.70 (17)
C9—C10—H10	120.0	C21—C22—C23	118.9 (2)
C11—C10—H10	120.0	C21—C22—H22	120.5
O1—C11—C2	124.87 (17)	C23—C22—H22	120.5
O1—C11—C10	113.6 (2)	C18—C23—C22	122.07 (18)
C2—C11—C10	121.5 (2)	C18—C23—H23	119.0
O1—C12—C13	111.22 (16)	C22—C23—H23	119.0
O1—C12—H12A	109.4	O2—C24—O3	123.72 (17)
C13—C12—H12A	109.4	O2—C24—C13	124.82 (16)
O1—C12—H12B	109.4	O3—C24—C13	111.43 (15)
C13—C12—H12B	109.4	O3—C25—H25A	109.5
H12A—C12—H12B	108.0	O3—C25—H25B	109.5
C12—C13—C1	109.08 (15)	H25A—C25—H25B	109.5
C12—C13—C24	112.97 (15)	O3—C25—H25C	109.5
C1—C13—C24	110.94 (14)	H25A—C25—H25C	109.5
C12—C13—C14	112.92 (14)	H25B—C25—H25C	109.5
C1—C13—C14	100.46 (13)		
			
C17—N1—C1—C2	−83.59 (19)	C2—C1—C13—C14	−166.96 (12)
C15—N1—C1—C2	154.79 (16)	C12—C13—C14—C18	53.3 (2)
C17—N1—C1—C13	156.33 (15)	C1—C13—C14—C18	169.33 (14)
C15—N1—C1—C13	34.71 (17)	C24—C13—C14—C18	−73.75 (18)
N1—C1—C2—C11	−94.1 (2)	C12—C13—C14—C15	−76.75 (19)
C13—C1—C2—C11	20.8 (2)	C1—C13—C14—C15	39.28 (16)
N1—C1—C2—C3	89.84 (19)	C24—C13—C14—C15	156.20 (14)
C13—C1—C2—C3	−155.34 (15)	C17—N1—C15—C14	−135.34 (16)
C11—C2—C3—C4	−173.48 (17)	C1—N1—C15—C14	−9.46 (19)
C1—C2—C3—C4	2.6 (3)	C17—N1—C15—C16	−13.4 (2)
C11—C2—C3—C8	5.5 (2)	C1—N1—C15—C16	112.51 (17)
C1—C2—C3—C8	−178.41 (15)	C18—C14—C15—N1	−149.27 (15)
C2—C3—C4—C5	−178.47 (18)	C13—C14—C15—N1	−18.92 (18)
C8—C3—C4—C5	2.6 (3)	C18—C14—C15—C16	90.68 (19)
C3—C4—C5—C6	−0.2 (3)	C13—C14—C15—C16	−138.98 (16)
C4—C5—C6—C7	−2.0 (4)	N1—C15—C16—S1	−18.99 (19)
C5—C6—C7—C8	1.7 (4)	C14—C15—C16—S1	98.69 (16)
C6—C7—C8—C9	−176.9 (2)	C17—S1—C16—C15	34.61 (15)
C6—C7—C8—C3	0.7 (3)	C15—N1—C17—S1	39.89 (18)
C4—C3—C8—C9	174.88 (19)	C1—N1—C17—S1	−81.16 (16)
C2—C3—C8—C9	−4.1 (3)	C16—S1—C17—N1	−44.10 (15)
C4—C3—C8—C7	−2.8 (3)	C15—C14—C18—C23	−138.51 (17)
C2—C3—C8—C7	178.22 (17)	C13—C14—C18—C23	98.24 (19)
C7—C8—C9—C10	177.1 (2)	C15—C14—C18—C19	41.0 (2)
C3—C8—C9—C10	−0.4 (3)	C13—C14—C18—C19	−82.2 (2)
C8—C9—C10—C11	3.6 (4)	C23—C18—C19—C20	2.5 (3)
C12—O1—C11—C2	12.4 (3)	C14—C18—C19—C20	−176.99 (19)
C12—O1—C11—C10	−170.33 (19)	C18—C19—C20—C21	−1.0 (3)
C3—C2—C11—O1	174.65 (18)	C19—C20—C21—C22	−1.3 (3)
C1—C2—C11—O1	−1.6 (3)	C19—C20—C21—Cl1	176.54 (18)
C3—C2—C11—C10	−2.4 (3)	C20—C21—C22—C23	1.9 (3)
C1—C2—C11—C10	−178.61 (18)	Cl1—C21—C22—C23	−175.99 (16)
C9—C10—C11—O1	−179.5 (2)	C19—C18—C23—C22	−2.0 (3)
C9—C10—C11—C2	−2.2 (3)	C14—C18—C23—C22	177.58 (17)
C11—O1—C12—C13	−41.6 (3)	C21—C22—C23—C18	−0.2 (3)
O1—C12—C13—C1	59.6 (2)	C25—O3—C24—O2	−0.4 (3)
O1—C12—C13—C24	−64.3 (2)	C25—O3—C24—C13	−178.41 (17)
O1—C12—C13—C14	170.33 (16)	C12—C13—C24—O2	149.37 (19)
N1—C1—C13—C12	73.74 (17)	C1—C13—C24—O2	26.5 (2)
C2—C1—C13—C12	−48.09 (18)	C14—C13—C24—O2	−83.6 (2)
N1—C1—C13—C24	−161.19 (13)	C12—C13—C24—O3	−32.6 (2)
C2—C1—C13—C24	76.98 (16)	C1—C13—C24—O3	−155.50 (15)
N1—C1—C13—C14	−45.13 (15)	C14—C13—C24—O3	94.37 (17)

### Hydrogen-bond geometry (Å, °)

**Table:**

Cg is the centroid of the chlorophenyl ring.

**Table:**

*D*—H···*A*	*D*—H	H···*A*	*D*···*A*	*D*—H···*A*
C12—H12B···N1	0.97	2.57	2.903 (3)	100
C25—H25A···Cg^i^	0.96	2.79	3.431 (3)	125

Symmetry codes: (i) *x*+1, *y*, *z*.
